# Does socioeconomic inequality occur in the multimorbidity among Brazilian adults?

**DOI:** 10.11606/s1518-8787.2020054002569

**Published:** 2020-11-27

**Authors:** Ândria Krolow Costa, Andréa Dâmaso Bertoldi, Andréia Turmina Fontanella, Luiz Roberto Ramos, Paulo Sergio Dourado Arrais, Vera Lucia Luiza, Sotero Serrate Mengue, Bruno Pereira Nunes

**Affiliations:** I Universidade Federal de Pelotas Faculdade de Enfermagem Programa de Pós-Graduação em Enfermagem PelotasRS Brasil Universidade Federal de Pelotas. Faculdade de Enfermagem. Programa de Pós-Graduação em Enfermagem. Pelotas, RS, Brasil; II Universidade Federal de Pelotas Faculdade de Medicina Departamento de Medicina Social PelotasRS Brasil Universidade Federal de Pelotas. Faculdade de Medicina. Departamento de Medicina Social. Programa de Pós-Graduação em Epidemiologia. Pelotas, RS, Brasil; III Universidade Federal do Rio Grande do Sul Faculdade de Medicina Programa de Pós-Graduação em Epidemiologia Porto AlegreRS Brasil Universidade Federal do Rio Grande do Sul. Faculdade de Medicina. Programa de Pós-Graduação em Epidemiologia. Porto Alegre, RS, Brasil; IV Universidade Federal de São Paulo Escola Paulista de Medicina Departamento de Medicina Preventiva São PauloSP Brasil Universidade Federal de São Paulo. Escola Paulista de Medicina. Departamento de Medicina Preventiva. São Paulo, SP, Brasil; V Universidade Federal do Ceará Faculdade de Farmácia, Odontologia e Enfermagem FortalezaCE Brasil Universidade Federal do Ceará. Faculdade de Farmácia, Odontologia e Enfermagem. Fortaleza, CE, Brasil; VI Fundação Oswaldo Cruz Escola Nacional de Saúde Pública Rio de JaneiroRJ Brasil Fundação Oswaldo Cruz. Escola Nacional de Saúde Pública. Rio de Janeiro, RJ, Brasil; VII Universidade Federal do Rio Grande do Sul Faculdade de Medicina Programa de Pós-Graduação em Epidemiologia Porto AlegreRS Brasil Universidade Federal do Rio Grande do Sul. Faculdade de Medicina. Programa de Pós-Graduação em Epidemiologia. Porto Alegre, RS, Brasil; VIII Universidade Federal de Pelotas Faculdade de Enfermagem Departamento de Enfermagem em Saúde Coletiva PelotasRS Brasil Universidade Federal de Pelotas. Faculdade de Enfermagem. Departamento de Enfermagem em Saúde Coletiva. Pelotas, RS, Brasil

**Keywords:** Adult, Socioeconomic Factors, Multimorbidity, Cross-Sectional Studies

## Abstract

**OBJECTIVE:**

To assess the prevalence of multimorbidity among Brazilian adults and its association with socioeconomic indicators.

**METHODS:**

Cross-sectional study that used data from the *Pesquisa Nacional Sobre Acesso, Utilização e Promoção do Uso Racional de Medicamentos no Brasil* (PNAUM – Brazilian National Survey on Access, Use and Promotion of Rational Use of Medicines), carried out between 2013 and 2014. The definition of multimorbidity was the coexistence, in a single individual, of two or more chronic diseases, measured through a list of 14 morbidities (self-reported medical diagnosis throughout life). Economic status and educational level were the socioeconomic indicators used, being the inequalities assessed through the Slope Index of Inequality (SII) and the Concentration Index, stratified by gender.

**RESULTS:**

The study comprehended 23,329 adults (52.8% of which were women), with an average age of 37.9 years. Hypertension and high cholesterol levels were the most prevalent conditions. The prevalence of multimorbidity was of 10.9% (95%CI 10.1–11.7) representing nearly 11 million individuals in Brazil, of which 14.5% (95%CI 13.5–15.4) were women and 6.8% (95%CI 5.9–7.8) were men. The occurrence of multimorbidity was similar according to the socioeconomic indicators. In the inequality analysis, we observed absolute and relative differences in men with a higher purchasing power (SII = 3.7; 95%CI 0.3–7.0) and higher educational level (CIX = 7.1; 95%CI 0.9–14.7), respectively.

**CONCLUSIONS:**

The frequency of comorbidities in Brazilian adults is high, especially in absolute terms. We only observed socioeconomic inequalities in multimorbidities among men.

## INTRODUCTION

The definition of multimorbidity is the coexistence of two or more chronic diseases in a single individual ^1^ . It has connections to a higher mortality risk; to a reduction in functional capacity, in cognitive skills and in quality of life; to the increase in usage of healthcare services and the amount of drugs prescribed per patient ^2^ .

The multimorbidity presents direct relation with the age increment ^3^^,^^4^ . In this sense, we observed a greater concentration of higher educational levels among the older adults (aged 60 years and older), with more scarce evidence among the younger population ^2^^,^^5^^,^^6^ .

A cohort study conducted in Australia emphasized the importance of observing multimorbidity in the younger ranges of the population. The study found that 4.4% (95%CI 3.4–5.7) of individuals aged between 20 and 39 years old had two or more chronic diseases, while in the population aged between 40 and 59 years old, the figure was of 15,0% (95%CI 13.1–17.2). ^7^ In Brazil, the multimorbidity is present in nearly one in every five and one in every 10 adults (≥ 18 years old) for ≥ 2 e ≥ 3, respectively. ^4^ A study conducted by Carvalho and contributors ^8^ showed that 5.6% of participants aged between 18 and 29 years old presented multimorbidity; this figure was of 12.3% for ages between 30 and 39 years old and of 23.9% between 40 and 49. Likewise, another study conducted in the country showed that between 18 and 24 years old, the percentage of multimorbidity was of 5.5%; between 24 and 44 years old, of 13.2%, and between 45 and 64 years old, of 36.2%. ^9^

The prevalence of multimorbidity is higher among women, according to research projects conducted in Brazil ^4^^,^^5^^,^^8^ , in Germany ^2^ and in Canada ^10^ . In addition, longitudinal analyses and a systematic review also observed such pattern.

Another factor usually associated with multimorbidity is the socioeconomic status – we observed a variation in the problem occurrence according to the socioeconomic characteristics of the population, depending on the socioeconomic indicator used. Certain studies evidenced an increase in multimorbidity as the educational level decreased ^2^^,^^4^^,^^5^^,^^8^^,^^12^ .

Systematic review and meta-analysis conducted in studies published up until 2014 evidenced that a low educational level was associated with a chance 1.64 (95%CI 1.41–1.91) times higher of multimorbidity in comparison to individuals with a higher educational level ^13^ . Compared with other socioeconomic indicators, especially income, the relation is less evident.

The results found in the literature regarding an association between multimorbidity and socioeconomic level are often heterogeneous. This may be due to the methodology used in the studies to assess the socioeconomic level, given that the literature describes several methods to assess it. Some of these are: assessment of household income ^13^ , educational level ^14^ , literacy, socioeconomic status, employment status, property ownership and even self-perception of poverty ^13^ . Of these methods, the educational level seems to be the indicator most strongly associated with multimorbidity. However, the literature on the subject presents analyses of inequalities considering the difference between the ends of this socioeconomic indicator.

To assess inequalities, more complex methods are under use. Among them, we call attention to the indexes that assess inequalities considering the entire stratification of the socioeconomic indicators, in addition to providing summary measures of the absolute and relative differences, which are methods more suited to the measurement of the inequalities and that we will use in the assessments proposed by this study.

In a study conducted with civil servants of a higher education institution in Rio de Janeiro, Brazil, Jantsch and contributors ^12^ found inverse relationship between educational level and multimorbidity, presenting three times more (RII = 2.97; 95%CI 1.94–4.54) occurrences between the ends of educational level. The indicator of absolute inequality (Slope Index of Inequality – SII) showed prevalence of multimorbidity 22 percentage points higher among women with lower educational levels than among those with higher educational levels.

The objective of this study was to assess the prevalence of multimorbidity and its association with socioeconomic indicators among Brazilian adults, considering the increase in multimorbidity among the younger age brackets of the population, the variation and the heterogeneous results according the socioeconomic levels of the individuals, the importance of assessing inequalities in health, and the scarcity of national studies. The hypothesis is that women with lower educational levels will present more multimorbidity, in terms of both absolute and relative inequalities.

## METHODS

Cross-sectional study, population-based, conducted on data originated from the population survey component of the *Pesquisa Nacional Sobre Acesso, Utilização e Promoção do Uso Racional de Medicamentos no Brasil* (PNAUM – Brazilian National Survey on Access, Use and Promotion of Rational Use of Medicines), conducted between 2013 and 2014.

The PNAUM interviewed 41,333 people in households of the urban areas in 245 municipalities of 26 states and the Federal District and had the goal of assessing the population's access to medicines, its use, rational use, sources of supply, and most prevalent morbidities that cause the use of medicines. After adjusting geopolitics region, gender and age, the sample represented nearly 171 million of people residing in the urban area of the country at the time. The sample draw considered three stages per cluster: municipality (primary sampling unit), census tract and household. The questionnaire, developed and tested by researchers from seven Brazilian universities, was conducted in a visit of the researcher to the interviewees' household. The questions asked covered, among several subjects, the current use of medicines to treat chronic diseases, and are available on the survey's website ^a^ . The survey used tablets for data collection, which were loaded into a software especially developed to the PNAUM. Further methodological detail is available on an article published by Sotero et al. ^16^

In this analysis, we considered adult individuals aged between 20 and 59 years old. The dependent variable was the presence of multimorbidity, characterized by the simultaneous occurrence of two or more chronic diseases ^17^ , measured through the report of medical diagnosis of the following morbidities: hypertension, high cholesterol level, depression, arthritis or rheumatism, diabetes, chronic pulmonary disease, coronary heart diseases, thyroid disorders, digestive disorder, stroke, neurological disorder, cancer, kidneys disease and other chronic diseases (for over six months). To other diseases, the survey kept only the conditions considered chronic, excluding morbidities and/or non-chronic conditions described by the interviewees (such as alcoholism and amygdalate). If the interviewee referred, in the question about other chronic diseases, any morbidity already inquired, the previous response was reviewed, accounting the morbidity only once.

The main exposure variable was socioeconomic status, assessed through two indicators: 1) economic status based on the property ownership (socioeconomic stratification in Brazil – ABEP 2013 ^b^ ), categorized in quintiles – due to the high proportion of middle-class individuals; 2) educational level of the interviewee, categorized in total years of education (none, 1–8, 9–11, ≥ 12). Other variables were gender (male/female) and age in years (20–29, 30–39, 40–49, 50–59). In the descriptive table of the sample, the socioeconomic status was presented as the status proposed by the indicator (A/B, C, D/E).

The prevalence estimates (%) of multimorbidity and the respective confidence intervals (95%) were calculated for the gender, age bracket, educational level and socioeconomic status. We based the assessment of the socioeconomic inequalities related to multimorbidity on the analysis of two indexes: 1) Slope Index of Inequality (SII), or absolute index of inequality; 2) Concentration Index of Inequality (CIX), or concentration index. The SII is used for stratification variables that are ordinal (such as socioeconomic status and educational level, used in the present analysis), representing the absolute difference in a health indicator among the most-advantaged and most-disadvantaged individuals and considering the entire distribution of the stratum. It also indicates the slope of the resulting regression line, being the absolute difference in the adjusted value of the health indicator between the two extremes of the socioeconomic indicator classification. The CIX considers all stratification categories, where 0 equals to equality18. In both indexes, we used the −100 to 100 scale to express the results.

We stratified our analysis by gender, and we weighted the results considering the study sampling design. We presented supplementary analyses for each of the country's geopolitical regions (North, Northeast, Midwest, Southeast and South). We considered the associations statistically significant when the 95%CI limits did not include the null value (SII or CIX equals to 0). We conducted all analyses using the Stata/SE 12.0 software.

The PNAUM was submitted to the National Commission for Ethics in Research (CONEP) under the Certificate of Presentation for Ethical Appreciation (CAAE) no. 18947013.6.0000.0008, and was approved under the report no. 398.131/2013, to be carried out at national level. The participants received information about the survey and signed an informed consent form before the interview.

## RESULTS

The study comprehended data from 23,329 adults (52.8% of which were women) that, when extrapolated to the target-population, represent nearly 96 million adults living in the urban area of Brazil. The total response rate varied from 43.9% of men aged between 20 and 39 years old to 53.4% of women aged between 20 and 39 years old. The sample mean age was of 37.9 years (38.3 years among women, 37.5 years among men).

In both genders, the age bracket ranging from 20 to 29 years and people with 1 to 8 educational years prevailed. The results among those who never attended school and those with 12 or over school years were also similar in both genders. Regarding the economic status, 24.8% felt under A/B classes and 20.1% felt under D/E classes ( Table 1 ).

**Table 1:** Sociodemographic characteristics, chronic diseases and multimorbidity per gender, among adults. PNAUM, Brazil, 2014.

Variable	Category	Female	Male
		% a	% a
	20–29	27.6	30.4
Age	30–39	26.9	26.6
	40–49	24.2	24.2
	50–59	21.3	18.8
	Never attended school	14.6	14.9
Educational level (in years of schooling)	1–8	42.8	44.2
	9–11	31.3	29.9
	≥ 12	11.3	11.0
	A/B	23.9	25.7
Socioeconomic status	C	56.0	54.0
	D/E	20.1	20.3
	Hypertension	18.9	11.8
	High cholesterol levels	8.5	5.6
	Depression	7.9	2.4
	Arthritis or rheumatism	6.0	2.2
	Diabetes	4.6	3.2
	Chronic pulmonar disease	4.2	1.9
Chronic disease b	Coronary heart diseases	3.3	2.2
	Thyroid disorders	1.9	0.2
	Digestive disorder	1.0	0.7
	Stroke	0.7	0.8
	Neurological disorder	0.7	1.0
	Cancer	0.3	0.0
	Kidney disease	0.3	0.3
	Another chronic disease	0.2	0.1
Multimorbidity	≥ 2 diseases	14.5	6.8

a Rates adjusted by sampling weight and by post-stratification according to age and gender.

b Diseases presented in ascending order of occurrence in women.

Regarding morbidities, we observed that hypertension and high cholesterol levels were the most prevalent diseases in both genders. Stomach disease, stroke, other neurological disorders, cancer, kidney diseases and other chronic diseases presented prevalence lower than 1.5% for both genders, and the last five, out of the entire set, had a lower prevalence among women than among men ( Table 1 ).

The presence of multimorbidity was 10.9% (95%CI 10.1–11.7), 14.5% (95%CI 13.5–15.4) among women and 6.8% (95%CI 5.9–7.8) among men ( Table 1 ). According to the age brackets, the prevalence varied from 2.7% (95%CI 2.2–4.4) among adults aged 20 to 29 years old to 26.9% (95%CI 25.2–28.7) among those aged 50 to 59 years old.

**Figure 1:**
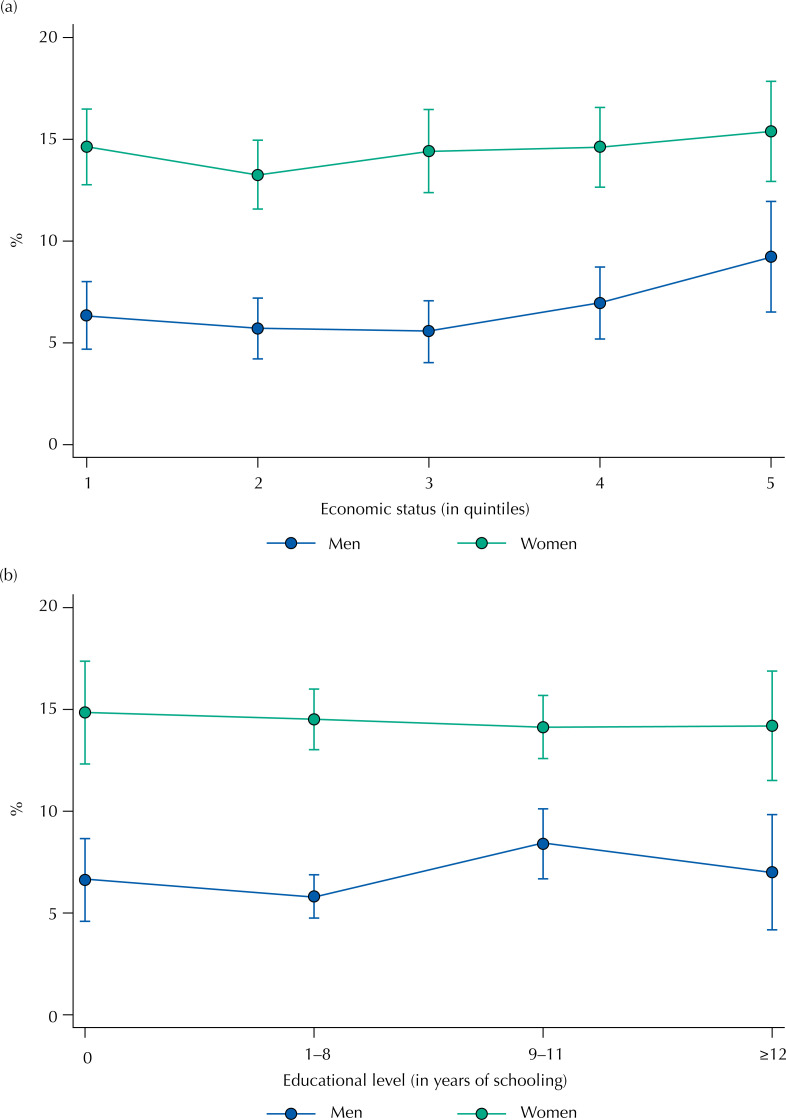
Multimorbidity per socioeconomic status and educational level, stratified by gender. PNAUM, Brazil, 2014.

The prevalence of multimorbidity was similar regarding the socioeconomic indicators, being a percentage higher among adults with 9 to 11 schooling years and belonging to the greatest quintile of property ownership ( Figure 1 ). We did not find results different than those of the general sample when conducting additional analyses stratified by age.

In the analyses of inequality, we observed a statistically significant difference among men. We found absolute and relative inequality, with the outcome concentrated among men with greater purchasing power (SI = 3.6 and SII = 9.5), and relative inequality, with the outcome concentrated among men with more school years (CIX = 7.1) ( Table 2 ).

**Table 2:** Multimorbidity inequalities analysis, stratified by gender and geopolitical regions. PNAUM, Brazil, 2014.

Regions	Indexes	Educational level		Socioeconomic status	
		Women	Men	Women	Men
North	SII (95%CI)	1.4 (-2.2 to 5.0)	-(-5.6 to 4.4)	-1.1 (-5.7 to 3.6)	2.8 (-2.4 to 8.0)
	CIX (95%CI)	1.4 (-6.3 to 9.1)	1.8 (-16.2 to 19.8)	-1.1 (-10.3 to 8.0)	8.8 (-7.5 to 25.2)
Northeast	SII (95%CI)	-2.3 (-7.6 to 3.0)	5.0 (-0.3 to 10.3)	0.9 (-3.6 to 5.4)	0.7 (-4.4 to 5.8)
	CIX (95%CI)	-2.5 (-9.2 to 4.1)	9.8 (-1.3 to 20.9)	0.2 (-5.8 to 6.2)	4.1 (-7.4 to 15.7)
Midwest	SII (95%CI)	-2.5 (-9.8 to 4.7)	-2.3 (-7.9; 3.1)	-3.9 (-10.9 to 3.0)	-1.4 (-8.9 to 6.1)
	CIX (95%CI)	-4.8 (-12.4 to 2.8)	-5.8 (-18.2 to 6.6)	-4.5 (-12.1 to 3.1)	-4.2 (-18.9 to 10.5)
Southeast	SII (95%CI)	0.6 (-5.4 to 6.7)	3.4 (-2.7 to 9.6)	3.2 (-2.3 to 8.7)	**7.3 (1.0 to 13.6)**
	CIX (95%CI)	1.1 (-4.7 −6.9)	10.3 (-3.1 to 23.7)	3.4 (-2.2 to 9.1)	**18.4 (5.8 to 31.0)**
South	SII (95%CI)	4.0 (-10.9 to 2.8)	0 (-5.4 to 5.2)	**-9.9 (-15.7 to −4.0)**	0.3 (-5.0 to 5.6)
	CIX (95%CI)	**-7.4 (-13.6 to −1.1)**	0.1 (-12.1 to 12.3)	**-10.6 (-16.4 to −4.7)**	1.0 (-11.5 to 13.4)
Brazil	SII (95%CI)	-0.89 (-4.1 to 234)	2.6 (-0.6 to 5.7)	1.4 (-1.6 to 4.4)	**3.6 (0.3 to 7.0)**
	CIX (95%CI)	-0.7 (-4.1 to 2.7)	**7.81 (0.01 to 14.1)**	1.4 (-2.0 to 4.8)	**9.5 (2.0 to 17.1)**

SII: absolute index of inequality; CIX: concentration index.

Bold figures represent statistically significant results.

We conducted supplementary analyses to assess the inequality of multimorbidity, stratified by gender, across the five geopolitical regions of Brazil ( Table 2 ). We observed statistically significant difference, considering the relative and absolute indexes of inequality, among men from the Southeast region with greater purchasing power (SII = 7.3 and CIX = 18.4) and among women from the South region with lower purchasing power (SII = −9.9 and CIX = −10.6). We also observed relative inequality among women with lower educational level.

**Figure 2:**
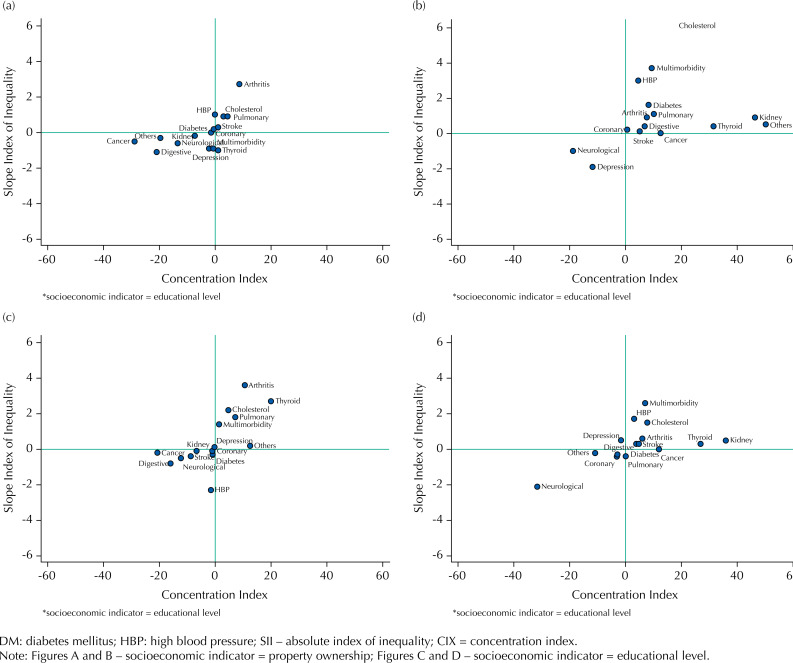
Analysis of inequalities of chronic diseases and multimorbidity, stratified by gender. PNAUM, Brazil, 2014.

In addition, we conducted inequalities analyses to isolated chronic diseases (statistic association not described in the figure). We observed absolute and relative inequality in both genders, with a disease pattern mostly concentrated among individuals with higher purchasing power, finding high cholesterol levels (men), arthritis (women) and thyroid (both). We also observed thyroid disorders more concentrated (CIX) among men with higher educational level. As for arthritis, we also verified absolute and relative inequality among women with higher educational level. We found that neurological disorders (men) were more concentrated (absolute and relative inequality) among those with lower educational level and, in relative manner, among those with lower purchasing power. We observed that digestive disorders (women) were most concentrated (absolute and relative inequality) among those with a lower educational level, in addition to a CIX with a health outcome concentrated among women with a lower purchasing power. To kidney disease, we observed a greater relative concentration among men with higher educational level and greater purchasing power. We found that cancer was most prevalent among women with lower educational levels (relative inequality) ( Figure 2 ).

## DISCUSSION

The multimorbidity affected one in every ten Brazilian adults. When we extrapolate to the country's population, this percentage represents nearly 11 million adults living in urban areas. In addition, we observed a greater prevalence of multimorbidity among women and older adults. We found social inequalities of low-magnitude (rates with significant difference, yet with values close to null) among men – relative inequality to educational level and absolute to socioeconomic class.

When we compare the occurrence of multimorbidity found in this study (10.9%) with literature, we observe a difference of roughly 18 percentage points. A recent systematic review and meta-analysis ^19^ also evidenced the occurrence of multimorbidity standardized by age affects 33.1% of the world population (adults and older adults); however, this rate varied across countries, being higher in those with higher incomes (37.9%) than in those countries with low and medium incomes (29.7%). In a cross-sectional study comprehending a sample of 28 countries with low and medium incomes, the multimorbidity was of 7.8%, presenting rates, respectively, of 3.8%, 12.8% and 21.3% to individuals aged between 18 to 49, 50 to 64 and ≥ 65 years old 19. In Brazil, national and local studies with adults (≥18 years old) found prevalence rates varying from 20 to 50% ^4^^,^^5^^,^^8^^,^^9^^,^^12^^,^^20^^,^^21^ , having three of them finding prevalence rates around 30%. In addition to the already mentioned limitations, two reasons may justify the differences regarding the results of the present work: 1) differences in the type, in the measurement method and in the number of diseases considered in each study. For example, a study conducted in São Paulo (between May, 2005 and May, 2007) used validated and accurate questionnaires for the measurement of mental disorders, increasing the identification of ocurrences ^21^ ; 2) national studies assessed the occurrence of multimorbidity in adults and older adults (≥ 60 years old), which tends to increase the average health outcome due to the higher occurrence among older adults.

Regarding age, our findings evidenced increased multimorbidity among older adults, varying from 2.7% (95%CI 2.2–4.4) to 26.9% (95%CI 25.2–28.7) to adults aged 20 to 29 and 50 to 59 years old, respectively. This association of multimorbidity with increased age is a common finding in studies, according to recent systemic review and metanalysis ^19^ .

However, although the age increase translates into greater frequency of multiple concomitant disorders, we emphasize that, in absolute figures (SII), multimorbidity is most common in adults 22. This finding confirms the need of action by the healthcare systems and services to address the concomitant disorders in this age bracket of the population, including both the prevention of multimorbidity and the prevention of new diseases in these adults that already present multimorbidity.

The present study also showed a prevalence of multimorbidity two times greater in women. A recent systematic revision and meta-analysis published on the subject ^19^ corroborate these findings, in which 21 of the 25 selected articles reported a greater prevalence of multimorbidity in women. In addition, in one of the articles featured in this revision, the multimorbidity was nearly two times greater in women (74% in women, 24% in men). ^19^ The study by Afshar et al. ^23^ also verified a higher occurrence of multimorbidity in the females in all countries considered in the study. A study ^4^ observed similar results in national level, showing a prevalence of two or more chronic diseases among women of 26.1% (95%CI 25.2–27.0) and of 17.5% (95%CI 16.6–18.3) among men. In addition, another study ^5^ brought results regarding the multimorbidity prevalence, showing 35.2% (95%CI 32.6–37.7) among women and 20.4% (95%CI 17.7–23.0) among men. A higher demand for healthcare services ^24^ may explain the higher prevalence of multimorbidity among women, with a greater exposition to diagnosis, as per the findings of a study carried out in the United States ^25^ , in which women aged between 18 and 64 years old were most likely to present multiple chronic disorders. In addition, men has a lower life expectancy and, therefore, a lower tendency to develop long-term health problems ^26^ . Thus, women, as they live longer, as more exposed throughout life to stressful events that compromise the physiopathological balance and favor the emerging of diseases. ^27^

We observed an association between multimorbidity and socioeconomic indicators only among men with higher purchasing power, when we assessed the absolute (SII) and the relative (CIX) inequality indexes, and among men with higher educational level, considering the relative inequality index (CIX). Despite the different measurement methods described in the literature, these results are in line with published evidence regarding such association ^2^^,^^10^^,^^22^ , including the national studies on educational levels ^4^^,^^5^^,^^8^ , whose results show inverse association between these indicators and the prevalence of morbidity. A study conducted by Afshar et al. ^23^ , in 28 low- and medium-income countries, found a positive relation, although not linear, between the country's gross domestic product and the prevalence of multimorbidity. Regarding the educational level, higher education was significantly associated with the reduction of the multimorbidity risk in the analyses of all regions.

The way the questionnaire was applied to the participants may explain the findings of this study, inquiring about the medical diagnosis regarding the assessed morbidities. It refers to individuals that, in theory, received medical attention and, thus, accessed to the diagnosis. In this sense, a differential error may exist in the assessed association: people with a higher purchasing power are more likely to access healthcare services and, consequently, get morbidities diagnosis. Thus, possibly, the prevalence of multimorbidity was higher among individuals with higher purchasing power (wealthier socioeconomic classes and higher educational level ^28^ . Yet, the analyses ran for isolated diseases evidenced inequalities, both for individuals with a higher purchasing power (high cholesterol levels, arthritis and thyroid disorders, other diseases) and for those with a lower purchasing power (digestive disorder) and lower educational level (neurological and digestive disorders and cancer), unlike the findings by Jantsch et al ^12^ , in which the morbidities, in general, were most prevalent among individuals with low educational level.

In the cross-sectional study aforementioned ^12^ , conducted in Rio de Janeiro, the association between education level and multimorbidity was different according to gender. Women who had not finished elementary school presented more than twice occurrences of multimorbidity than those who had finished postgrad. However, among men, the multimorbidity was lower among those who had finished and not finished the elementary school, but this result was not statistically significant. In the calculation of the SII, the study observed that women with a lower educational level presented 22% more multimorbidity than those with higher educational levels. In relative terms, using the CIX, the study found a morbidity three times higher in lower educational levels, with a greater inequality level among women ^12^ .

The systematic review and the meta-analysis published by Pathirana et al. ^13^ , with the aim to assess this evaluation, showed heterogeneous methods of measuring the socioeconomic level. The review verified that a low educational level was associated with a 64% increased chance of multimorbidity occurrence. However, when aggregating these studies by age, the association is higher in the older populations. This fact may explain why we did not find such association in this study, given that the focus were adults aged between 20 and 59 years old.

We verified socioeconomic inequalities for certain diseases in Isolation. Although presenting a list of diseases slightly different than those adopted in this work, Jantsch et at. ^12^ found that all conditions (hypertension, diabetes, dyslipidemia, coronary heart disease, stroke, chronic pulmonary disease, peptic ulcer disease, cholecystitis, osteomuscular disorder and thyroid disorder), except repetitive strain injury, showed a significant linear trend among women, more prevalent among those with low educational levels. Among men, the association was statistically significant only for hypertension, diabetes, coronary heart disease, repetitive strain injury and peptic ulcer disease. The low rate of prevalence of certain disorders in this group may also explain the lack of association among men, such as thyroid disorders and cancer.

To enable a better comprehension of this finding, certain limitations must be emphasized. First, the limited number of chronic diseases considered in the study may underestimate the occurrence of concomitant morbidities, reducing the percentage of individuals presenting multimorbidity. Even though the questionnaire listed a question inquiring about another chronic disease, the interviewee may have failed to mention another existing disease, once that the question was not specific about the morbidities – which would rely on the interviewee's memory. Second, the information about the presence of a chronic disease was based in the self-reported medical diagnosis, being this an usual method in epidemiological studies, but with limited accuracy ^29^ , even for prevalent conditions ^21^^,^^4^ . Third, the lack of standards in the definition of the problem hampers the definition of multimorbidity cases and the comparison between studies ^30^ .

The results show, in general, socioeconomic equality in the occurrence of multimorbidity, measured by the medical diagnosis of diseases. The low association found is a result discrepant from existing literature on the subject and demands further analyses able to support the health public policies regarding to inequalities. It is also noteworthy the importance of using these indexes in the inequality analyses, given that they consider the entire sample distribution of the indicator, and not only end values of it, as it is the case in raw analyses of income and educational levels ^15^ .

Nevertheless, the relative frequency and, mostly, the absolute frequency of multimorbidity, in this own, emphasize the relevance of multimorbidity among Brazilian adults, especially among women. With the trend of emergence of multimorbidity in earlier stages of life, there is an increase in the odds of an individual seek for healthcare services (especially emergency and hospitalization services), of premature death and worse quality of life. Therefore, it is important to direct public policies to serve this parcel of the population, who is getting sick increasingly early.

## Footnotes

a Pesquisa Nacional sobre Acesso, Utilização e Promoção do Uso Racional de Medicamentos no Brasil. 2013. [cited 2020 Apr 20]. Available from: http://www.ufrgs.br/pnaum .

b Associação Brasileira de Empresas de Pesquisa. Critério Brasil de Classificação Econômica. São Paulo: Abep, 2013.
