# Supplementary material for: Does socioeconomic inequality occur in the multimorbidity among Brazilian adults?
Source: Rev Saude Publica. 2020 Nov 27;54:138. doi: 10.11606/s1518-8787.2020054002569 (PMC7703529; doi:10.11606/s1518-8787.2020054002569)
Supplement: Translation

# Translation: Existe desigualdade socioeconômica na multimorbidade entre adultos brasileiros?

**Authors:** Ândria Krolow Costa, Andréa Dâmaso Bertoldi, Andréia Turmina Fontanella, Luiz Roberto Ramos, Paulo Sergio Dourado Arrais, Vera Lucia Luiza, Sotero Serrate Mengue, Bruno Pereira Nunes

**Affiliations:** I PelotasRS Brasil Universidade Federal de Pelotas. Faculdade de Enfermagem. Programa de Pós-Graduação em Enfermagem. Pelotas, RS, Brasil; II PelotasRS Brasil Universidade Federal de Pelotas. Faculdade de Medicina. Departamento de Medicina Social. Programa de Pós-Graduação em Epidemiologia. Pelotas, RS, Brasil; III Porto AlegreRS Brasil Universidade Federal do Rio Grande do Sul. Faculdade de Medicina. Programa de Pós-Graduação em Epidemiologia. Porto Alegre, RS, Brasil; IV São PauloSP Brasil Universidade Federal de São Paulo. Escola Paulista de Medicina. Departamento de Medicina Preventiva. São Paulo, SP, Brasil; V FortalezaCE Brasil Universidade Federal do Ceará. Faculdade de Farmácia, Odontologia e Enfermagem. Fortaleza, CE, Brasil; VI Rio de JaneiroRJ Brasil Fundação Oswaldo Cruz. Escola Nacional de Saúde Pública. Rio de Janeiro, RJ, Brasil; VII Porto AlegreRS Brasil Universidade Federal do Rio Grande do Sul. Faculdade de Medicina. Programa de Pós-Graduação em Epidemiologia. Porto Alegre, RS, Brasil; VIII PelotasRS Brasil Universidade Federal de Pelotas. Faculdade de Enfermagem. Departamento de Enfermagem em Saúde Coletiva. Pelotas, RS, Brasil

**Keywords:** Adulto, Fatores Socioeconômicos, Multimorbidade, Estudos transversais

## Abstract

**OBJETIVO:**

Avaliar a prevalência de multimorbidade e a associação desta com indicadores socioeconômicos entre adultos brasileiros.

**MÉTODOS:**

Estudo transversal que utilizou dados oriundos da Pesquisa Nacional sobre Acesso, Utilização e Promoção do Uso Racional de Medicamentos no Brasil, realizada entre 2013 e 2014. Multimorbidade foi definida como a coexistência, no mesmo indivíduo, de duas ou mais doenças crônicas, e é mensurada a partir de uma lista de 14 morbidades (autorrelato de diagnóstico médico na vida). Classe econômica e escolaridade foram os indicadores socioeconômicos utilizados, sendo as desigualdades avaliadas pelo *Slope Index of Inequality* (SII) e pelo *Concentration Index* (CIX), estratificadas por sexo.

**RESULTADOS:**

O estudo considerou 23.329 mil adultos (52,8% de mulheres), com média de idade de 37,9 anos. Hipertensão e colesterol alto foram as condições mais prevalentes. A prevalência de multimorbidade foi de 10,9% (IC95% 10,1–11,7), representando, aproximadamente, 11 milhões de indivíduos no Brasil, sendo 14,5% (IC95% 13,5–15,4) entre mulheres e 6,8% (IC95% 5,9–7,8) entre homens. A ocorrência de multimorbidade foi similar segundo os indicadores socioeconômicos. Nas análises de desigualdade, observou-se diferença absoluta e relativa para homens com maior poder aquisitivo (SII = 3,7; IC95% 0,3–7,0) e maior escolaridade (CIX = 7,1; IC95% 0,9–14,7), respectivamente.

**CONCLUSÕES:**

A frequência de adultos brasileiros com multimorbidade é alta, principalmente em termos absolutos. Desigualdades socioeconômicas na multimorbidade foram observadas somente entre homens.

## INTRODUÇÃO

Multimorbidade pode ser definida como a coexistência de duas ou mais doenças crônicas em um mesmo indivíduo ^1^ . Está relacionada com maior risco de mortalidade; com redução da capacidade funcional, habilidades cognitivas e qualidade de vida; com o aumento do uso de serviços de saúde e do número de medicamentos prescritos por paciente ^2^ .

A multimorbidade apresenta relação direta com o aumento da idade ^3^^,^^4^ . Nesse sentido, observa-se maior concentração de estudos com idosos (população maior de 60 anos), com evidências mais escassas entre a população mais jovem ^2^^,^^5^^,^^6^ .

Um estudo de coorte realizado na Austrália destacou a importância de observar a multimorbidade em faixas etárias mais jovens da população, uma vez que 4,4% (IC95% 3,4–5,7) das pessoas acompanhadas no estudo, com idade entre 20 e 39 anos, tinham duas ou mais doenças crônicas, e, para a população entre 40 e 59 anos, este número foi de 15,0% (IC95% 13,1–17,2) ^7^ . No Brasil, a multimorbidade está presente, aproximadamente, em um a cada cinco e um a cada 10 adultos (≥ 18 anos) para ≥ 2 e ≥ 3 morbidades, respectivamente ^4^ . Estudo realizado por Carvalho e colaboradores ^8^ mostrou que 5,6% dos participantes com idade entre 18 e 29 anos tinham multimorbidade; este percentual foi de 12,3% para idades entre 30 e 39 anos e de 23,9%, entre 40 e 49 anos. Da mesma forma, outro estudo conduzido no país mostrou que entre 18 e 24 anos o percentual de multimorbidade foi de 5,5%; entre 25 e 44 anos, 13,2%; e entre 45 e 64 anos, 36,2% ^9^ .

A prevalência de multimorbidade é maior entre mulheres, conforme evidenciado por estudos realizados no Brasil ^4^^,^^5^^,^^8^ , na Alemanha ^2^ e no Canadá ^10^ . Análises longitudinais ^11^ e uma revisão sistemática ^6^ também observaram esse padrão.

Outro fator normalmente associado a multimorbidade é o nível socioeconômico – observa-se variação da ocorrência do problema de acordo com as características socioeconômicas da população, segundo o indicador socioeconômico utilizado. Estudos evidenciaram aumento da multimorbidade com a diminuição do nível educacional ^2^^,^^4^^,^^5^^,^^8^^,^^12^ .

Revisão sistemática e meta-análise com estudos publicados até 2014 evidenciaram que baixa escolaridade estava associada a 1,64 (IC95% 1,41–1,91) vezes mais chances de multimorbidade em comparação com indivíduos com alta escolaridade ^13^ . Com outros indicadores socioeconômicos, principalmente renda, a relação é menos evidente.

Os resultados encontrados na literatura sobre associação de multimorbidade e nível socioeconômico muitas vezes são heterogêneos. Isso pode ocorrer devido à metodologia utilizada nos estudos para avaliação do nível socioeconômico, uma vez que diversas são as formas descritas na literatura para essa mensuração. Algumas dessas técnicas são: avaliação da renda familiar ^13^ , nível educacional ^14^ , alfabetização, classe social, situação de emprego, posse de bens e até mesmo autopercepção de pobreza ^13^ . Desses métodos, o nível educacional parece ser o indicador mais fortemente associado a multimorbidade. Porém, a literatura sobre o tema traz análises de desigualdades considerando a diferença entre os extremos deste indicador socioeconômico.

Métodos mais complexos de avaliação das desigualdades vêm sendo utilizados. Dentre esses, destacam-se os índices que avaliam as desigualdades considerando toda a estratificação dos indicadores socioeconômicos, além de fornecerem medidas síntese das diferenças absolutas e relativas ^15^ , métodos mais apropriados para a mensuração de desigualdades e que serão utilizados nas avaliações propostas por este trabalho.

No Brasil, Jantsch e colaboradores ^12^ , em um estudo com servidores de uma instituição de ensino superior do Rio de Janeiro, observaram tendência inversa entre escolaridade e multimorbidade, com três vezes mais (RII = 2,97; IC95% 1,94–4.54) desfechos entre os extremos de escolaridade. O indicador de desigualdade absoluta ( *Slope Index of Inequality* – SII) mostrou prevalência de multimorbidade 22 pontos percentuais maior entre mulheres com menor nível educacional do que entre aquelas com nível educacional maior.

Considerando o aumento da multimorbidade entre a faixa etária mais jovem da população, a variação e os resultados heterogêneos segundo níveis socioeconômicos dos indivíduos, a importância de avaliar desigualdades em saúde e a escassez de estudos nacionais, o objetivo deste estudo foi avaliar a prevalência de multimorbidade e a associação desta com indicadores socioeconômicos entre adultos brasileiros. A hipótese é que mulheres de menor escolaridade apresentarão mais multimorbidade, em termos de desigualdades tanto absolutas como relativas.

## MÉTODOS

Estudo transversal, de base populacional, realizado a partir de dados oriundos do componente inquérito populacional da Pesquisa Nacional Sobre Acesso, Utilização e Promoção do Uso Racional de Medicamentos no Brasil (PNAUM), realizada entre setembro de 2013 e fevereiro de 2014

A PNAUM envolveu entrevistas com 41.433 mil moradores residentes em domicílios da zona urbana de 245 municípios das 26 unidades da Federação e no Distrito Federal, e objetivou avaliar o acesso da população a medicamentos, sua utilização, uso racional, fontes de obtenção e morbidades mais prevalentes que levam ao uso de medicamentos. Após ajustes para região geopolítica, sexo e idade, a amostra representa aproximadamente 171 milhões de pessoas residentes na zona urbana do país à época. O sorteio da amostra considerou três estágios por conglomerado: município (unidade amostral primária), setor censitário e domicílio. O questionário, desenvolvido e testado por pesquisadores de sete universidades do Brasil, foi aplicado em uma visita do entrevistador à residência do entrevistado. As perguntas realizadas tratavam, entre diferentes temas, sobre uso atual de medicamentos para doenças crônicas e encontram-se disponíveis no sítio da pesquisa a . Os dados coletados foram registrados em *tablets* , por um *software* desenvolvido especificamente para a PNAUM. Mais detalhes metodológicos podem ser obtidos no artigo publicado por Sotero et al. ^16^

Para esta análise, foram considerados indivíduos adultos com idade entre 20 e 59 anos. A variável dependente deste estudo foi a presença de multimorbidade, avaliada a partir da ocorrência simultânea de duas ou mais doenças crônicas ^17^ , medidas pelo relato do diagnóstico médico das seguintes morbidades: hipertensão, colesterol alto, depressão, artrite ou reumatismo, diabetes, doença pulmonar crônica, doenças do coração, tireoide, doença gástrica, AVC (acidente vascular cerebral), doença neurológica, câncer, doença renal e outra doença crônica (com mais de seis meses de duração). Para outra doença crônica, foram mantidas somente as condições consideradas crônicas, excluindo morbidades e/ou condições não crônicas descritas pelos entrevistados (como alcoolismo, amidalite). Caso o entrevistado tivesse referido, na pergunta sobre outra doença crônica, alguma morbidade já questionada (como hipertensão), foi revisada a resposta anterior considerando a morbidade apenas uma vez.

A principal variável de exposição foi nível socioeconômico, avaliado com base em dois indicadores: 1) classe econômica baseada na posse de bens (classificação econômica Brasil – ABEP 2013 b ), categorizada em quintis – devido à alta proporção de indivíduos na classe C; 2) nível de escolaridade do entrevistado, categorizada em anos completos de estudo (não estudou, 1–8, 9–11, ≥ 12). Outras variáveis utilizadas foram sexo (masculino/feminino) e idade em anos completos (20–29, 30–39, 40–49, 50–59). Na tabela descritiva da amostra, a variável classe econômica foi apresentada nas classes propostas pelo indicador (A-B, C, D-E).

As estimativas de prevalência (%) de multimorbidade e os respectivos intervalos de confiança (95%) foram calculados para as variáveis sexo, faixa etária, escolaridade e classe econômica. A avaliação das desigualdades socioeconômicas relacionadas a multimorbidade foi realizada a partir da análise de dois índices: 1) *Slope Index of Inequality* (SII), ou índice absoluto de desigualdade; 2) *Concentration Index* (CIX), ou índice de concentração. O SII é utilizado para variáveis de estratificação que sejam ordinais (como classificação econômica e escolaridade, usadas na presente análise), representando a diferença absoluta de um indicador de saúde entre os indivíduos mais favorecidos e menos favorecidos e considerando toda a distribuição do estratificador. Indica ainda a inclinação da linha de regressão resultante, sendo a diferença absoluta no valor ajustado do indicador de saúde entre os valores mais alto e mais baixo da classificação do indicador socioeconômico. O CIX considera todas as categorias da variável de estratificação, sendo 0 a igualdade ^18^ . Para ambos os índices, utilizou-se a escala de −100 a 100 para expressar os resultados.

As análises realizadas foram estratificadas por sexo, e os percentuais foram ponderados considerando o desenho amostral do estudo. Análises suplementares das desigualdades foram apresentadas por região geopolítica do país (Norte, Nordeste, Centro-Oeste, Sudeste e Sul). Associações foram consideradas estatisticamente significativas quando os limites do IC95% não compreenderam a nulidade (SII ou CIX igual a 0). Todas as análises foram realizadas no *software* Stata/SE 12.0.

A PNAUM foi submetida à Comissão Nacional de Ética em Pesquisa (Conep), sob o Certificado de Apresentação para Apreciação Ética (CAAE) 18947013.6.0000.0008, e foi aprovada mediante o Parecer n° 398.131/2013, para execução em âmbito nacional. Os participantes foram esclarecidos sobre a pesquisa, e as entrevistas foram precedidas pela assinatura do termo de consentimento livre e esclarecido (TCLE).

## RESULTADOS

O estudo incluiu informações de 23.329 adultos (52,8% de mulheres), que extrapolando para a população-alvo, representam, aproximadamente, 96 milhões de adultos moradores da zona urbana do Brasil. A taxa de resposta total variou de 49,3%, para homens de 20 a 39 anos de idade, a 53,4%, para mulheres de 20 a 39 anos. A média de idade da amostra entrevistada foi de 37,9 anos (38,3 entre mulheres e 37,5 entre homens).

Em ambos os sexos prevaleceram a faixa etária 20–29 anos e pessoas com escolaridade entre 1 e 8 anos. O resultado entre os que nunca estudaram e os que tinham 12 anos ou mais de estudo também foi similar entre os sexos. Em relação à classe econômica, 24,8% eram da classe A/B e 20,1% da classe D/E ( Tabela 1 ).

**Tabela 1:** Características sociodemográficas, doenças crônicas e multimorbidade, segundo o sexo entre adultos. PNAUM, Brasil, 2014.

Variável	Categoria	Sexo feminino (n = 15.386)	Sexo masculino (n = 7.943)
		% a	% a
	20–29	27,6	30,4
Idade	30–39	26,9	26,6
	40–49	24,2	24,2
	50–59	21,3	18,8
	Não estudou	14,6	14,9
Escolaridade (em anos completos de estudo)	1–8	42,8	44,2
	9–11	31,3	29,9
	≥ 12	11,3	11,0
	A/B	23,9	25,7
Classe econômica	C	56,0	54,0
	D/E	20,1	20,3
	Hipertensão	18,9	11,8
	Colesterol alto	8,5	5,6
	Depressão	7,9	2,4
	Artrite ou reumatismo	6,0	2,2
	Diabetes	4,6	3,2
	Doença pulmonar crônica	4,2	1,9
Doenças crônicas b	Doenças do coração	3,3	2,2
	Tireoide	1,9	0,2
	Doença gástrica	1,0	0,7
	Acidente vascular cerebral (AVC)	0,7	0,8
	Doença neurológica	0,7	1,0
	Câncer	0,3	0,0
	Doença renal	0,3	0,3
	Outra doença crônica	0,2	0,1
Multimorbidade	≥ 2 doenças	14,5	6,8

a Percentuais ajustados pelos pesos amostrais e por pós-estratificação segundo idade e sexo.

b Doenças apresentadas em ordem decrescente de ocorrência no sexo feminino.

Quanto a morbidades, observou-se que hipertensão e colesterol alto foram as doenças mais prevalentes em ambos os sexos. Doença gástrica, AVC, outras doenças neurológicas, câncer, doença renal e outras doenças crônicas apresentaram prevalência menor que 1,5% para ambos os sexos, e apenas as últimas cinco, de todo o conjunto, tiveram prevalência menor entre mulheres do que entre homens ( Tabela 1 ).

A presença de multimorbidade foi de 10,9% (IC95% 10,1–11,7), sendo 14,5% (IC95% 13,5–15,4) entre mulheres e 6,8% (IC95% 5,9–7,8) entre homens ( Tabela 1 ). Segundo os estratos de idade, a prevalência variou de 2,7% (IC95% 2,2–4,4) para adultos entre 20 e 29 anos a 26,9% (IC95% 25,2–28,7) para aqueles com 50–59 anos.

A prevalência de multimorbidade foi similar de acordo com os indicadores socioeconômicos, sendo percentualmente maior entre adultos com 9–11 anos de estudo e pertencentes ao maior quintil da posse de bens ( Figura 1 ). Análises adicionais estratificadas por idade não encontraram resultado diferente do observado para a amostra geral.

**Figura 1:** Multimorbidade segundo classe econômica e escolaridade, estratificado por sexo. PNAUM, Brasil, 2014.

Nas análises de desigualdade, observou-se diferença estatisticamente significativa para homens. Verificaram-se desigualdade absoluta e relativa, com um padrão de desfecho que se concentra entre homens com maior poder aquisitivo (SII = 3,6 e CIX = 9,5), e desigualdade relativa, com um padrão de desfecho que se concentra entre homens com maior escolaridade (CIX = 7,1) ( Tabela 2 ).

**Tabela 2:** Análise de desigualdades da multimorbidade, estratificado por sexo e regiões geopolíticas. PNAUM, Brasil, 2014.

Regiões	Índices	Escolaridade		Classe econômica	
		Mulheres	Homens	Mulheres	Homens
Norte	SII (IC95%)	1,4 (-2,2 a 5,0)	-(-5,6 a 4,4)	-1,1 (-5,7 a 3,6)	2,8 (-2,4 a 8,0)
	CIX (IC95%)	1,4 (-6,3 a 9,1)	1,8 (-16,2 a 19,8)	-1,1 (-10,3 a 8,0)	8,8 (-7,5 a 25,2)
Nordeste	SII (IC95%)	-2,3 (-7,6 a 3,0)	5,0 (-0,3 a 10,3)	0,9 (-3,6 a 5,4)	0,7 (-4,4 a 5,8)
	CIX (IC95%)	-2,5 (-9,2 a 4,1)	9,8 (-1,3 a 20,9)	0,2 (-5,8 a 6,2)	4,1 (-7,4 a 15,7)
Centro-Oeste	SII (IC95%)	-2,5 (-9,8 a 4,7)	-2,3 (-7,9; 3,1)	-3,9 (-10,9 a 3,0)	-1,4 (-8,9 a 6,1)
	CIX (IC95%)	-4,8 (-12,4 a 2,8)	-5,8 (-18,2 a 6,6)	-4,5 (-12,1 a 3,1)	-4,2 (-18,9 a 10,5)
Sudeste	SII (IC95%)	0,6 (-5,4 a 6,7)	3,4 (-2,7 a 9,6)	3,2 (-2,3 a 8,7)	**7,3 (1,0 a 13,6)**
	CIX (IC95%)	1,1 (-4,7 −6,9)	10,3 (-3,1 a 23,7)	3,4 (-2,2 a 9,1)	**18,4 (5,8 a 31,0)**
Sul	SII (IC95%)	4,0 (-10,9 a 2,8)	0 (-5,4 a 5,2)	**-9,9 (-15,7 a −4,0)**	0,3 (-5,0 a 5,6)
	CIX (IC95%)	**-7,4 (-13,6 a −1,1)**	0,1 (-12,1 a 12,3)	**-10,6 (-16,4 a −4,7)**	1,0 (-11,5 a 13,4)
Brasil	SII (IC95%)	-0,89 (-4,1 a 234)	2,6 (-0,6 a 5,7)	1,4 (-1,6 a 4,4)	**3,6 (0,3 a 7,0)**
	CIX (IC95%)	-0,7 (-4,1 a 2,7)	**7,81 (0,01 a 14,1)**	1,4 (-2,0 a 4,8)	**9,5 (2,0 a 17,1)**

SII – índice absoluto de desigualdade; CIX – índice de concentração.

Valores em negrito indicam resultados estatisticamente significativos.

Foram executadas análises suplementares para avaliar a desigualdade da multimorbidade, estratificada por sexo, entre as cinco regiões geopolíticas do Brasil ( Tabela 2 ). Observou-se diferença estatisticamente significativa, considerando os índices relativo e absoluto de desigualdades, para homens da região Sudeste com maior poder aquisitivo (SII = 7,3 e CIX = 18,4) e para mulheres com menor poder aquisitivo na região Sul (SII = −9,9 e CIX = −10,6). Também para o sexo feminino foi observada desigualdade relativa (CIX = −7,4) entre as menos escolarizadas.

Também foram realizadas análises de desigualdade para doenças crônicas isoladas (associação estatística não descrita na figura). Observou-se desigualdade absoluta e relativa, em ambos os sexos, com um padrão de doenças mais concentrado entre indivíduos com maior poder aquisitivo para colesterol (homens), artrite (mulheres) e tireoide (ambos). Problemas na tireoide também foram observados de forma mais concentrada (CIX) entre homens de maior escolaridade. Para artrite, no sexo feminino também se verificou desigualdade absoluta e relativa a favor das mais escolarizadas. Doenças neurológicas (homens) foram mais concentradas (desigualdade absoluta e relativa) para os menos escolarizados e de forma relativa para os de menor poder aquisitivo. Doenças gástricas (mulheres) foram mais concentradas (desigualdade absoluta e relativa) entre as menos escolarizadas, além de um CIX com padrão de desfecho concentrando-se entre mulheres de menor poder aquisitivo. Para doença renal, verificou-se maior concentração relativa entre homens mais escolarizados e de maior poder aquisitivo. Câncer foi mais prevalente entre mulheres menos escolarizadas (desigualdade relativa) ( Figura 2 ).

**Figura 2:** Análise das desigualdades das doenças crônicas e multimorbidade, estratificado por sexo. PNAUM, Brasil, 2014.

## DISCUSSÃO

Multimorbidade acometeu um a cada dez adultos brasileiros. Extrapolando para a população do país, esse percentual representa, aproximadamente, 11 milhões de adultos residentes em áreas urbanas. Além disso, verificou-se maior prevalência de multimorbidade entre mulheres e adultos mais velhos. Desigualdades sociais de baixa magnitude (índices com diferença significativa, mas com valores próximos a nulidade) foram observadas entre homens – desigualdade relativa para escolaridade e absoluta para classe econômica.

Ao compararmos a ocorrência de multimorbidade encontrada neste estudo (10,9%) com a literatura, observa-se diferença de 18 pontos percentuais aproximadamente. Recente revisão sistemática e meta-análise ^19^ evidenciou também que a ocorrência de multimorbidade padronizada por idade afeta 33,1% da população mundial (adultos e idosos), porém este percentual variou entre os países, sendo maior daqueles de alta renda (37,9%) do que nos de baixa e média renda (29,7%). Já em estudo transversal composto por uma amostra de 28 países de baixa e média renda, a multimorbidade foi de 7,8%, sendo, respectivamente, de 3,8%, 12,8% e 21,3% para pessoas com idade de 18–49, 50–64 e ≥ 65 anos ^19^ . No Brasil, estudos nacionais e locais com adultos (≥ 18 anos) encontraram prevalência variando de, aproximadamente, 20% a 50% ^4^^,^^5^^,^^8^^,^^9^^,^^12^^,^^20^^,^^21^ , com três destes encontrando prevalência ao redor de 30%. Além das limitações já citadas, duas explicações podem justificar as diferenças com o resultado do presente trabalho: 1) diferença no tipo, na forma de mensuração e no número de doenças consideradas em cada estudo. Por exemplo, estudo realizado em São Paulo (entre maio de 2005 e maio de 2007) utilizou questionários validados e acurados para a mensuração dos problemas de saúde mental, aumentando a identificação de casos ^21^ ; 2) estudos nacionais avaliaram a ocorrência de multimorbidade em adultos e idosos (≥ 60 anos), o que tende a aumentar a média do desfecho devido à maior ocorrência entre pessoas mais velhas.

Em relação à idade, os achados evidenciaram aumento da multimorbidade entre adultos mais velhos, variando de 2,7% (IC95% 2,2–4,4) a 26,9% (IC95% 25,2–28,7) para adultos de 20–29 e 50–59 anos, respectivamente. Esta associação de multimorbidade com aumento da idade é um achado comum nos estudos, conforme evidenciado por recente revisão sistemática e meta-análise ^19^ . Porém, apesar do aumento da idade significar maior frequência de múltiplos problemas concomitantes, destaca-se que, em números absolutos (SII), a multimorbidade é mais comum em adultos ^22^ , fato que ratifica a necessidade de ações dos sistemas e serviços de saúde para o manejo dos problemas concomitantes nesta faixa etária da população, incluindo tanto a prevenção da multimorbidade quanto a prevenção de novas doenças para esses adultos que já possuem multimorbidade.

O presente estudo também mostrou prevalência de multimorbidade duas vezes maior entre mulheres. Esses achados são corroborados por recente revisão sistemática e meta-análise publicada sobre o assunto ^19^ , em que 21 dos 25 artigos selecionados relataram maior prevalência de multimorbidade entre o sexo feminino. Além disso, em um dos artigos trazidos por esta revisão, a multimorbidade era quase o dobro nas mulheres (74% e 26% nos homens) ^19^ . O estudo de Afshar et al. ^23^ também verificou maior ocorrência de multimorbidade no sexo feminino em todos os países considerados no estudo. Resultados similares em nível nacional foram observados em estudo ^4^ que mostrou que a prevalência de duas ou mais doenças crônicas entre mulheres foi de 26,1% (IC95% 25,2–27,0) e entre homens foi de 17,5% (IC95% 16,6–18,3). Outro estudo ^5^ trouxe resultados semelhantes quanto à prevalência de multimorbidade, sendo de 35,2% (IC95% 32,6–37,7) entre mulheres e de 20,4% (IC95% 17,7–23,0) entre homens. Essa prevalência maior de multimorbidade entre o sexo feminino pode ser explicada pela maior procura por serviços de saúde ^24^ , com maior exposição ao recebimento de diagnóstico de doenças, conforme evidenciado em estudo conduzido nos Estados Unidos ^25^ , no qual mulheres com idade entre 18 e 64 anos foram mais propensas do que homens a ter múltiplas condições crônicas. Além disso, homens têm menor expectativa de vida e, assim, menor tendência ao desenvolvimento de problemas de saúde de maior duração ^26^ . Desta forma, mulheres, por viverem mais, são mais expostas ao longo da vida a eventos estressantes que comprometem o equilíbrio fisiopatológico e favorecem o desenvolvimento de doenças ^27^ .

Foi observada associação entre multimorbidade e indicadores de nível socioeconômico apenas para homens com maior poder aquisitivo, quando avaliado o índice de desigualdade absoluta (SII) e relativo (CIX), e para homens com maior escolaridade, considerando o índice de desigualdade relativa (CIX). Mesmo com diferentes formas de mensuração descritas na literatura, este resultado vai de encontro a evidências já publicadas sobre tal associação ^2^^,^^10^^,^^22^ , incluindo os estudos nacionais sobre escolaridade ^4^^,^^5^^,^^8^ , cujos resultados mostram associação inversa entre esses indicadores e a prevalência de multimorbidade. Estudo realizado por Afshar et al. ^23^ , em 28 países de baixa e média renda, encontrou relação positiva, mas não linear, entre o Produto Interno Bruto do país e a prevalência de multimorbidade. Quanto à escolaridade, ensino superior foi significativamente associado à diminuição do risco de multimorbidade nas análises de todas as regiões.

Os achados do presente estudo podem ser explicados pela forma como o questionário foi aplicado aos participantes, em que foi perguntado sobre o diagnóstico médico para as morbidades avaliadas. Este fato considera indivíduos que, teoricamente, passaram por atendimento médico e, assim, conseguiram acesso ao diagnóstico. Neste sentido, pode existir erro diferencial na associação avaliada: pessoas com maior poder aquisitivo tendem a ter maior acesso a serviços de saúde e, consequentemente, ao diagnóstico de morbidades. Dessa forma, possivelmente, a prevalência de multimorbidade foi maior entre indivíduos com maior poder aquisitivo (classes econômicas mais ricas e maior nível de escolaridade) do que entre pessoas com menor poder aquisitivo ^28^ . Por outro lado, as análises executadas para as doenças de forma isolada evidenciaram desigualdades, tanto para pessoas com maior poder aquisitivo (colesterol, artrite e tireoide, outras doenças) quanto para aquelas com menor poder aquisitivo (doença gástrica) e menos escolarizadas (doenças neurológicas, gástricas e câncer), diferentemente do encontrado por Jantsch et al. ^12^ , em que as morbidades, de modo geral, foram mais prevalentes entre pessoas com baixo nível educacional.

No estudo transversal acima mencionado ^12^ , realizado no Rio de Janeiro, a associação entre escolaridade e multimorbidade diferiu entre os sexos. Mulheres com ensino fundamental incompleto apresentaram mais do dobro de ocorrência de multimorbidade do que as que haviam realizado pós-graduação. Por outro lado, para homens, a multimorbidade foi menor entre aqueles com ensino fundamental incompleto e completo, mas este resultado não foi estatisticamente significativo. Para o cálculo do SII, verificou-se que mulheres com menor nível educacional tinham 22% mais multimorbidade do que aquelas com mais educação formal. Em termos relativos, utilizando o CIX, verificou-se presença de multimorbidade três vezes maior em níveis educacionais menores, com maior nível de desigualdade entre mulheres ^12^ .

A revisão sistemática e meta-análise publicada por Pathirana et al. ^13^ , com o objetivo de avaliar esta associação, mostrou formas heterogêneas de avaliação do nível socioeconômico. Verificou-se que baixa escolaridade estava associada a uma chance aumentada em 64% de ocorrência de multimorbidade. Porém, quando esses estudos foram agrupados de acordo com idade, a associação foi maior nas populações mais velhas. Este fato pode explicar por que não foi verificada, na presente análise, esta associação, uma vez que o foco foram adultos entre 20 e 59 anos.

Foram verificadas desigualdades socioeconômicas para algumas doenças de forma isolada. Embora com uma lista de doenças um pouco diferente da adotada por este trabalho, Jantsch et al. ^12^ verificaram que todas as condições (hipertensão, diabetes, dislipidemia, doença cardíaca coronária, acidente vascular encefálico, condição pulmonar, úlcera péptica, colecistite, doença osteomuscular e doença da tireoide), exceto lesões por esforço repetitivo, mostraram tendência linear significativa entre mulheres, mais prevalentes entre aquelas com níveis de escolaridade mais baixos. Para homens, a associação apenas foi estatisticamente significante para hipertensão, diabetes, doença cardíaca coronária, lesão por esforço repetitivo e úlcera péptica. A falta de associação entre homens, encontrada neste estudo, pode também ser explicada pela baixa prevalência de alguns problemas nesse grupo, como, por exemplo, tireoide e câncer.

Para melhor compreensão dos achados, algumas limitações devem ser destacadas. Primeiro, o número limitado de doenças crônicas consideradas no estudo pode subestimar a ocorrência de morbidades concomitantes, reduzindo o percentual de pessoas com multimorbidade. Mesmo havendo no questionário a pergunta sobre outra doença crônica, o entrevistado pode não ter citado outra possível doença existente, já que a pergunta não foi específica para as morbidades – o que pode depender da memória do entrevistado. Segundo, a informação sobre presença de doença crônica foi baseada no diagnóstico médico autorreferido, sendo este um método usual em estudos epidemiológicos, porém com acurácia limitada ^29^ , mesmo para condições prevalentes ^21^^,^^4^ . Terceiro, a falta de padronização na definição do problema dificulta a definição de casos de multimorbidade e a comparação entre os estudos ^30^ .

Os resultados indicam, de maneira geral, igualdade socioeconômica na ocorrência de multimorbidade, medida pelo diagnóstico médico de doenças. A baixa associação verificada é um resultado discrepante da literatura existente sobre o tema e necessita de mais análises que possam subsidiar as políticas públicas de saúde no que se refere a desigualdades. Cabe ressaltar também a importância de usar esses índices nas análises de desigualdades, uma vez que consideram toda a distribuição amostral do indicador, e não apenas valores extremos deste, como ocorre em análises brutas de renda e escolaridade ^15^ .

Não obstante, a frequência relativa e, principalmente, a frequência absoluta de multimorbidade, por si só, destacam a relevância da multimorbidade entre adultos brasileiros, principalmente mulheres. Com a tendência do aparecimento da multimorbidade em fases mais jovens da vida, aumenta a probabilidade de indivíduos usarem serviços (principalmente de urgência e emergência e hospitalização), de morte prematura e pior qualidade de vida. É, assim, importante o direcionamento de políticas públicas que atendam esta parcela da população que está adoecendo cada vez mais cedo.

## Footnotes

a Pesquisa Nacional sobre Acesso, Utilização e Promoção do Uso Racional de Medicamentos no Brasil. 2013. [citado 20 abr 2020]. Disponível em: http://www.ufrgs.br/pnaum .

b Associação Brasileira de Empresas de Pesquisa. Critério Brasil de Classificação Econômica. São Paulo: Abep, 2013.
